# Adolescents’ screen time exposure and the associated physical and psychological factors: an urban–rural perspective

**DOI:** 10.3389/fpubh.2026.1908142

**Published:** 2026-10-02

**Authors:** Gauri A. Oka, Ashish S. Ranade, Mayur K. Shinde, Madhuri P. Kasabekar, Prasad D. Pore

**Affiliations:** 1 Central Research and Publication Unit, Bharati Vidyapeeth Deemed University Medical College, Pune, India; 2 Blooming Buds Centre for Pediatric Orthopaedics, Deenanath Mangeshkar Hospital and Research Centre, Pune, India; 3 Department of Community Medicine, Bharati Vidyapeeth Deemed University Medical College, Pune, India

**Keywords:** adolescents, physical, psychological, rural, screen time, urban

## Abstract

**Background:**

Data exist on adolescents’ screen time behavior, but are lacking on urban–rural differences. We compared daily screen time exposures and associated anthropometric parameters, frequency of outdoor physical activity, and psychological parameters of urban and rural school-going adolescents.

**Methods:**

This was a cross-sectional study. Using five districts of one of the administrative divisions of a western Indian state as the sampling frame, the first two urban and first two rural schools were selected from a randomly reordered list of schools. We included 627 students from 5th, 7th, and 9th standards as representatives of the adolescent age group (10–19 years). Self-reported outdoor physical activity, psychological assessment using the Strengths and Difficulties Questionnaire (SDQ) and daily time spent on each were recorded. We determined associations between area of residence, frequency of outdoor physical activity, and SDQ scores and screen time using the chi-square test or Fisher’s exact test. Multivariable regression analysis was used to determine the predictors of excessive screen time (>2 h/day). Adjusted odds ratios (aOR) and 95% confidence intervals (CI) were calculated.

**Results:**

There were 334 (53.3%) urban and 293 (46.7%) rural students. The mean age was 12.5 ± 1.4 years. Mean daily total screen time was 111.9 ± 71.7 min. Urban compared to rural [121.5 ± 75.9 min vs. 100.9 ± 65.0 min; *p* = 0.001] and boys compared to girls [122.9 ± 81.9 min vs. 100.9 ± 58.0 min; *p* < 0.000] had longer total screen time exposure. Being a rural resident was significantly associated with a >2 days/week of outdoor physical activity [78.8% (rural) vs. 35.9% (urban), respectively; OR 6.64, 95% CI 4.64–9.51, *p* < 0.000]. Students with daily screen time of >2 h had some/high needs on the SDQ hyperactivity scale [11.4% vs. 5.7%; *p* = 0.022]. Males had significantly higher odds of >2 h of total daily screen time than females (aOR = 1.51, 95% CI 1.05–2.17; *p* = 0.026).

**Conclusion:**

The associations between longer screen time exposure and worse physical and psychological factors warrant interventions with special attention to urban–rural differences.

## Background

Smartphones, tablets, and laptops, and the digital content on streaming platforms and social media applications have rapidly transformed the daily lives of young people, influencing their communication, learning, and recreation. While the associated educational and social benefits are undeniable, the amount of time adolescents spend in front of screens has raised concerns among clinicians, public health researchers, and policymakers worldwide (1). International health authorities, including the World Health Organization (WHO) (2) and the American Academy of Pediatrics (AAP) (3), as well as the Indian Academy of Paediatrics (IAP) (4), have issued guidelines recommending screen time limits for children. There are no explicit guidelines on screen time exposure limits for adolescents. However, evidence generated from several studies worldwide has shown that in adolescents, daily screen time exceeding 2 h is associated with physical and psychological problems (5, 6). The Health Behavior in School-Aged Children (HBSC) report on school-going adolescents aged 11, 13, and 15 years from across Europe, Central Asia, and Canada found that a substantial proportion of adolescents engaged in more than 2 h of screen time on a typical school day, with higher exposure documented among older adolescents, boys, and those from lower socioeconomic backgrounds (7). According to the Pew Research Center, more than 90% of adolescents used social networking sites, with half of them being “almost constantly” connected to social networks (8). Excessive screen time is associated with an increased risk of functional, cognitive, behavioral, and emotional disorders (9). It is also associated with decreased self-esteem and an increase in mental health problems in young adults (6, 10). A large Chinese study has shown that University students who spent more than 2 h per day on screens were not only at greater risk of experiencing emotional and behavioral symptoms, but also social adaptation difficulties (11). Khan et al. observed that more than 1 h per day of screen time was associated with adverse mental health effects in adolescents. Furthermore, increasing screen time exposure was negatively associated with life satisfaction levels and positively associated with psychosomatic complaints in a dose-dependent manner (12). In a meta-analysis of 57,831 individuals less than 20 years of age, Yue et al. found positive correlations between daily computer use, mobile use and TV watching and the risk of low back pain separately, with a dose-dependent association between low back pain and daily computer use (13).

The relationship between duration of screen time exposure and overweight and obesity has also received considerable attention from researchers. Excessive sedentary screen time is usually at the expense of outdoor physical activity and is associated with poor eating habits (14). Studies have shown associations between longer screen time and higher body mass index (BMI) in adolescents, with effect sizes varying across study settings (14, 15). Rapid urbanization in India has led to a wider digital penetration and easy and affordable access to digital devices, particularly smartphones, even in rural areas. Although screen time duration among adolescents has been the subject of several studies in India (16–18), there are limited data on the linkages between BMI, frequency of outdoor physical activity, and psychological parameters and screen time duration as well as urban–rural differences, if any (5, 6). Understanding urban–rural differences in screen time behaviors of adolescents and also in the associated factors will aid in the development of school-based interventions tailored to the specific geographical setting. Against this backdrop, the objective of the present study was to determine screen time exposure and its associations with BMI, frequency of outdoor activity, and psychological parameters among adolescent students, with a focus on urban–rural differences.

## Methods

Using a cross-sectional design, we analyzed our pre-intervention baseline data, generated under the “SanSKAR” project (Screen time reduction and Surya Namaskars—a comprehensive intervention for nonspecific back pain in adolescents: a study protocol) (19). The study is registered with the Clinical Trial Registry of India (CTRI/2024/07/070522). Approval from the Institutional Ethics Committee and permissions from the respective school principals were obtained before starting the study. Informed parental consent and written assent from students were obtained before initiating data collection.

The sampling frame was five districts (Pune, Satara, Sangli, Kolhapur and Solapur) from the Pune Division of the state of Maharashtra, India. Schools from these districts were stratified into two separate lists in Microsoft Excel: urban and rural. According to the study protocol, it was predecided that Microsoft Excel’s random number generation command would be used to reorder the names of these urban and rural schools. The randomly reordered list would be followed chronologically for seeking permissions from schools. If we failed to obtain permission from a school, we would approach the next school in the list. Thus, we approached the first two urban schools from Pune district and the first two rural schools, one each from Satara and Sangli districts, for permission to participate. All four schools permitted us. Hence, we conducted the study in these four schools. During our first school visit, we distributed child assent forms, parental consent forms, and information sheets detailing the study procedures amongst the students for parental use. We conducted detailed discussions with the respective class teachers. After allowing 8–10 days for receipt of parental consent and child assent, the date for the baseline data collection was set. The participant enrolment flowchart (Figure 1) depicts the number of students enrolled at baseline from each class/the number of students who provided both assent and parental consent/the number of students who were approached for participation from each class in the two urban and two rural schools. The data were collected between July and August 2024 in two schools and between June and August 2025 in the other two schools, thus ensuring seasonal uniformity and similar school timetables across the sites. We enrolled 627 students from 5th, 7th, and 9th grades as representatives of the adolescent age group, that is, 10–19 years.

**Figure 1:**
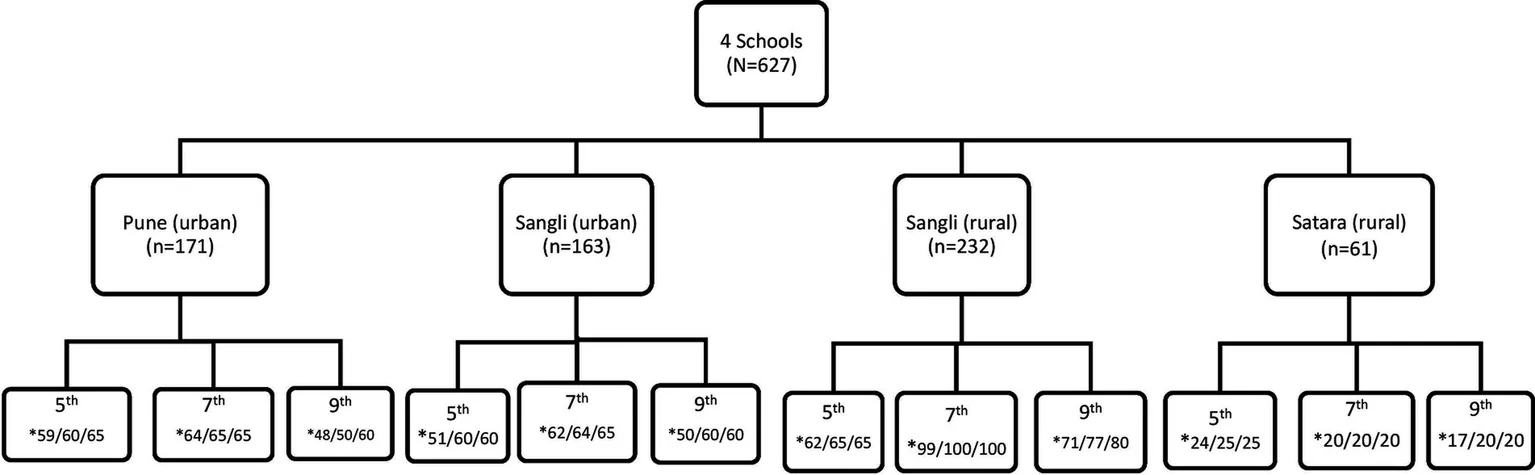
Participant enrolment flowchart.

Sociodemographic (name, age, sex, grade, urban/rural residence) and anthropometric data (height, weight, and BMI) were noted. The students’ heights and weights were measured using a calibrated stadiometer with an accuracy of up to 0.1 cm. The students were requested to stand without shoes and socks for their height measurement. The head was tilted in the Frankfurt plane, and the reading was noted. A digital weighing machine with an accuracy of 0.01 g was used for measuring the weight. Students were asked to stand barefoot on the machine in their school uniform without shoes or socks, and without any extra clothing such as sweaters or jackets. The body mass index (BMI) was calculated as weight (kg)/height (m^2^). The students’ BMIs were categorized using the cut-offs published by Khadilkar et al. (20). Data were also collected on students’ frequency of outdoor physical activity at school, defined as the number of days per week engaged in outdoor physical activity such as field sports, dance, swimming, etc. All the schools had strict rules prohibiting students from bringing any digital devices to the campus. Information was collected on the types of digital devices used at home, along with self-reported average daily screen time per device based on a week’s recall. For this study, a daily screen time of more than 2 h was defined as excessive screen time. The Strengths and Difficulties Questionnaire (SDQ) (21) was used for psychological assessment. According to the authors and creators of the SDQ, the SDQ is a genuinely dimensional measure of child mental health. Children with higher total difficulty scores have greater psychopathology as judged by the prevalence of clinical disorder. This was found to be true cross-sectionally and also in predicting the disorder status 3 years later (22). The SDQ is a validated instrument for identifying adolescents’ emotional and behavioral problems with a good internal consistency (Cronbach’s alpha 0.75), and sensitivity and specificity of 0.75 and 0.90, respectively (23). It is a 25-item questionnaire comprising five scales and five items per scale. The scales are emotional problems, conduct problems, hyperactivity, peer problems, and the prosocial scale. Each item in the scale has three options to choose from, namely, “not true,” “somewhat true,” and “certainly true.” For each of the five scales, the scores can range from 0 to 10. The total difficulties score for each adolescent is calculated by adding the scores on all the scales except the prosocial scale. The newer four-band categorization was employed for classifying the SDQ scores into “close to average”, “slightly raised (or slightly lowered for prosocial score)”, “high (or low for prosocial score)”, and “very high or (very low for prosocial score)”. The need for psychological support in each scale is categorized based on the adolescent’s individual scale scores into “low need”, “some need”, or “high need” according to the pre-specified cut-offs for that scale (24). The data collection forms and the SDQ were distributed in the classrooms and completed by the students under the supervision of a study team member. Clarifications and assistance with understanding and filling in the forms were provided as and when necessary.

## Data analysis

A power analysis was conducted to determine the statistical power of comparing the total screen time between urban and rural groups. The achieved sample size of 627 adolescent students (334 urban and 293 rural) provided 95.5% power to detect a difference in daily total screen time at a 5% level of significance. Data were analyzed using SPSS version 28 for Windows package (IBM SPSS Statistics for Windows, Version 28.0. Armonk, NY: IBM Corp). Frequencies and proportions were calculated for categorical variables. For normally distributed data, means with standard deviations (SD), and for non-normally distributed data, medians with interquartile ranges (IQR; Q1, Q3) were calculated for continuous variables. The independent *t*-test was used to compare means of normally distributed variables, and the Mann–Whitney *U*-test was used to compare the medians of non-normally distributed variables. Associations were determined using the chi-square test or Fisher’s exact test as appropriate. Multivariable regression analysis was used to determine the significant predictors of excessive screen time (>2 h/day). Adjusted odds ratios (aOR) and 95% confidence intervals (CI) were calculated, and a *p*-value <0.05 was considered statistically significant.

## Results

Of the 627 participating students, 334 (53.3%) were urban residents. There were 314 (50.1%) girls. The mean age of the students was 12.5 ± 1.4 years (range 9.5–15.6 years). Eight rural students (one boy, seven girls) and four urban students (two boys, two girls) reported not using any digital device. These students were included in the analysis, and their screen time was zero minutes/day. The mean daily screen time of the study sample was 111.9 ± 71.7 min. It was found that 172/627 (27.4%) students spent more than 2 h/day across all devices (combined). The types of devices used were mobile phones [538 (85.8%)], television (TV) [491 (78.3%)], and computers/laptops [221 (35.2%)]. There was no statistically significant difference between the mean ages of students with more than 2 h of daily total screen time exposure as compared to ≤2 h (12.7 ± 1.4 years and 12.5 ± 1.4 years, respectively; *p* = 0.093). The participant characteristics (*N* = 627) are depicted in Table 1.

**Table 1:** Participant characteristics.

Variable	Mean (SD)
Age (years)	12.5 (1.4)
BMI (kg/m^2^)	17.8 (3.6)
Total screen time (min/day)	111.9 (71.7)

Variable	Category	*n* (%)
Sex	Female	314 (50.1)
	Male	313 (49.9)
Residence	Rural	293 (46.7)
	Urban	334 (53.3)
BMI category	Underweight	426 (67.9)
	Normal	135 (21.5)
	Overweight	40 (6.4)
	Obese	26 (4.1)
Computer use	Yes	221 (35.2)
	No	406 (64.8)
TV use	Yes	491 (78.3)
	No	136 (21.7)
Mobile phone use	Yes	538 (85.8)
	No	89 (14.2)
Total daily screen time	≤2 h/day	455 (72.6)
	>2 h/day	172 (27.4)
Outdoor physical activity frequency	≤Twice/week	276 (44.1)
	>Twice/week	351 (55.9)

The differences in device usage between males and females, and between urban and rural students, are depicted in Table 2.

**Table 2:** Male–female and urban–rural differences in digital device use.

Comparator	Mobile phone *n* (%)	OR (95% CI)	TV *n* (%)	OR (95% CI)	Computer/laptop *n* (%)	OR (95% CI)
Sex						
Male (*n* = 313)	283 (90.4)	2.18 (1.36–3.49)	242 (77.3)	0.89 (0.60–1.30)	111 (35.5)	1.01 (0.73–1.41)
Female (*n* = 314)	255 (81.2)		249 (79.3)		110 (35.0)	
Residence						
Urban (*n* = 334)	304 (91.0)	2.55 (1.59–4.09)	270 (80.8)	1.37 (0.93–2.01)	118 (35.3)	1.00 (0.72–1.39)
Rural (*n* = 293)	234 (79.9)		221 (75.4)		103 (35.2)	

According to Table 2, a significantly greater proportion of male students used mobile phones [283 (90.4%), OR 2.18, 95% CI 1.36–3.49]. Also, a greater proportion of urban students used mobile phones than rural students [304 (91.0%) vs. 234 (79.9%); OR 2.55, 95% CI 1.59–4.09]. There were no statistically significant male–female and urban–rural differences in the use of other devices.

A significantly greater proportion of urban students had >2 h of screen time exposure than rural students (31.1% vs. 23.2%; *p* = 0.026). It was found that boys spent a significantly longer time on mobile phones as compared to girls [60 (30, 60) min vs. 30 (15, 60) min; *p* < 0.001] and also had significantly longer median daily total screen time exposure [120 (60, 180) min vs. 90 (60, 120) min; *p* < 0.004]. Urban compared with rural students had longer median screen time exposures across all devices, including total screen time. The differences were significant for mobile phone duration [50 (30, 60) min vs. 30 (15, 60) min; *p* = 0.005], TV viewing [45 (30, 60) min vs. 30 (5, 60) min; *p* = 0.017], and total screen time duration [120 (60, 150) min vs. 90 (60, 120) min; *p* < 0.001] (Table 3).

**Table 3:** Male–female and urban–rural differences in daily screen time durations.

Comparator	Mobile phone mean (SD) Median [Q1, Q3]	TV mean (SD) Median [Q1, Q3]	Computer/laptop mean (SD) Median [Q1, Q3]	Total screen time mean (SD) Median [Q1, Q3]
Sex				
Male (*n* = 313)	56.6 (45.4) 60 [30, 60]	49.1 (46.3) 60 [15, 60]	17.2 (32.4) 0 [0, 30]	122.9 (81.9) 120 [60, 180]
Female (*n* = 314)	39.8 (32.8) 30 [15, 60]	43.4 (35.0) 30 [15, 60]	17.7 (30.7) 0 [0, 30]	100.9 (58.0) 90 [60, 120]
*p* value	<0.001	0.463	0.766	0.004
Residence				
Urban (*n* = 334)	53.1 (43.1) 50 [30, 60]	49.9 (43.8) 45 [30, 60]	18.6 (34.9) 0 [0, 30]	121.5 (75.9) 120 [60, 150]
Rural (*n* = 293)	42.6 (36.5) 30 [15, 60]	42.0 (37.6) 30 [5, 60]	16.2 (27.2) 0 [0, 30]	100.9 (65.0) 90 [60, 120]
*p* value	**0.005**	**0.017**	0.92	**<0.001**

Bold *p*-values indicate statistical significance (*p* < 0.05).

The mean BMI of urban students was greater than that of rural students (18.1 ± 3.6 kg/m^2^ vs. 17.4 ± 3.6 kg/m^2^, respectively; *p* = 0.023). A significantly greater proportion of students with >2 h of daily screen time exposure were overweight and obese as compared to those with ≤2 h of exposure [14.5% vs. 9.0%, respectively; OR 1.61, 95% CI 1.01–2.56; *p* = 0.044. Being a rural resident was significantly associated with a higher frequency (>2 days/week) of outdoor physical activity than their urban counterparts (78.8% vs. 35.9%, respectively; OR 6.64, 95% CI 4.64–9.51, *p* < 0.000). Although the association was not statistically significant, students with >2 h of daily total screen time were likely to engage in ≤2 days a week of outdoor physical activity as compared with those with ≤2 h of daily screen time [50.0% vs. 41.8%; OR 1.39, 95% CI 0.98–1.98].

A greater proportion of students with >2 h of daily screen time exposure had some/high needs across all SDQ scales compared to those with ≤2 h, with the difference being statistically significant in the hyperactivity scale (11.4% vs. 5.7%; *p* = 0.022) (Table 4).

**Table 4:** Differences in the proportion of students with some/high needs on all SDQ scales according to their screen time exposure.

Screen time	Students with some/high needs (%)					
	Emotional problems	Conduct problems	Hyperactivity	Peer problems	Prosocial	Total difficulties^*^
≤2 h	12.0	19.8	5.7	25.6	9.2	60.9
>2 h	14.5	24.7	11.4	30.1	11.5	62.8
*p* value	0.411	0.214	**0.022**	0.303	0.444	0.713

^*^Sum of all scales except “prosocial.” Bold *p*-values indicate statistical significance (*p* < 0.05).

Multivariable logistic regression analysis (Table 5) was performed to determine the independent predictors of excessive screen time, and it was found to be statistically significant. The model revealed that male students had significantly higher odds of >2 h of total daily screen time exposure than females (aOR = 1.51, 95% CI 1.05–2.17; *p* = 0.026). No statistically significant associations were observed with age, place of residence, frequency of sports, or BMI category.

**Table 5:** Multivariable logistic regression for predictors of >2 h of total daily screen time.

Variable	Category	aOR (95% CI)	*p* value
Age	Continuous	1.06 (0.93–1.22)	0.361
Sex	Female	1	**0.026**
	Male	1.51 (1.05–2.17)	
Residence	Rural	1	0.174
	Urban	1.33 (0.88–2.00)	
Outdoor physical activity	>Twice a week	1	0.435
	≤Twice a week	1.18 (0.78–1.78)	
BMI category	Normal/underweight	1	0.087
	Overweight/obese	1.61 (0.93–2.78)	

Bold *p*-values indicate statistical significance (*p* < 0.05).

(*p* > 0.05 for all).

Figure 2 depicts differences in the characteristics of urban and rural students with >2 h of daily screen time exposure. It was observed that among all the students with screen time exposure of >2 h (*n* = 172), the urban areas had significantly greater proportion of males (66.3% vs. 45.6%; *p* = 0.007), students with ≤2 days/week of outdoor physical activity (66.3% vs. 25.0%; *p* < 0.001), and students with some/high needs across all SDQ scales except the prosocial score [EP: 20.4% vs. 6.0%, *p* < 0.001; CP: 38.5% vs. 4.5%, *p* = 0.01; HP: 19.0% vs. 0.0%, *p* < 0.001; PP: 44.4% vs. 9.0%, *p* < 0.001; total difficulties: 89.4% vs. 22.1%, *p* < 0.001].

**Figure 2:**
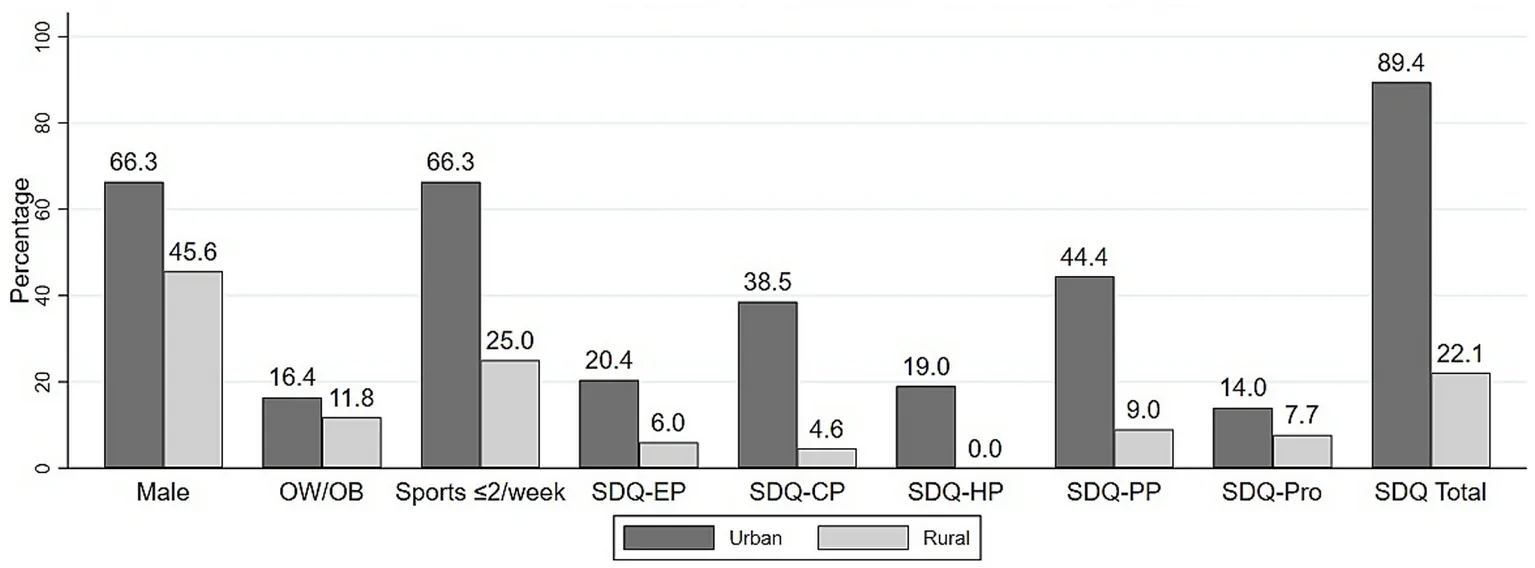
Urban–rural comparison of characteristics of students with >2 h of total daily screen time exposure.

## Discussion

Growing urbanization and the rapid advancement and expansion of digital technology have facilitated early and easy access to smartphones and other digital devices even in the rural areas of India today. Our study objectives were to compare screen time exposure among adolescent students in urban and rural schools and to determine associations between screen time and three important parameters, namely, BMI, frequency of outdoor physical activity, and psychological factors.

We found that a greater proportion of urban students had excess screen time and a significantly longer total daily screen time exposure than rural students. Chandran et al., in their study comparing screen time exposure among urban and rural high school students in Karnataka, found that the total daily screen time of urban students was more than twice that of rural students (25). We also found that being a male student was an independent predictor of more than 2 h of daily screen time exposure. Our findings align with those reported by Imrani et al., Wu et al., and Cui et al., who found that boys and urban residents had significantly higher odds of spending more time on screens than girls and rural students (26–28). This is expected given the different lifestyles of urban and rural adolescents in India, especially because of their different domestic environments and family structures (6). In rural India, the social structure is predominantly shaped by traditional norms, customs, and values. Village life is characterized by close-knit communities where everyone knows each other’s social status, family background, and personal histories. Rural life primarily revolves around the extended family. For various familial and social reasons, rural children in India perform domestic work such as washing utensils and clothes, fetching water from considerable distances, and working on farms (29). The urban family structure has increasingly shifted to nuclear units. This transformation is largely driven by economic pressures, space constraints, and changing aspirations. These social and institutional differences play a significant role in shaping the lifestyle of the younger generation (30). Consequently, adolescents from urban families differ from rural families in their lifestyles, occupational patterns, social relationships, value systems, and access to resources. Most urban areas offer greater availability and easy access to infrastructure and public leisure spaces such as sports clubs, open grounds, gymnasiums and parks. Despite this, rural adolescents exhibit healthier habits than urban ones, with greater preference for active leisure activities, shorter sitting time, and less daily screen time (31). The most widely used online platforms among urban and rural adolescents in India include Instagram, YouTube, WhatsApp, Twitter, LinkedIn, and Snapchat (32–34). With the ever-increasing social media presence of the young population enabled by easy access to the internet and digital devices, there are emerging parental challenges to foster digital health while maintaining their growing children’s healthy physical and mental habits. The challenges faced by parents in India are further compounded by varying cultural expectations and diverse socioeconomic scenarios, influenced by the nuclear or extended-family structures (34). There are no legal age restrictions on social media or smartphone use for children and adolescents in India. However, students are prohibited from bringing mobile phones to schools across India.

Many studies from around the world have reported associations between excessive screen time exposure and adverse physical health among adolescents (35, 36). In their study of 1952 US adolescents, Zablotsky et al. found significant associations between daily screen time of four or more hours and infrequent physical activity and having weight-related concerns (35). A systematic review of reviews by Stiglic et al. has found the strongest evidence for associations between longer screen time and adiposity and unhealthy diet (14). The association between longer daily screen time exposure and overweight and obesity has been established in several studies (15, 37, 38). Our urban students had a higher BMI than their rural counterparts. We also found that a significantly greater proportion of students with more than 2 h of daily screen time exposure were overweight and obese as compared to those with less than 2 h of exposure. Similar findings have been reported by Asut et al. (36) and Brodersen et al. (37). It is well known that adolescents nowadays engage less in outdoor physical activity and spend much of their waking hours on mobile phones, laptops, and television (35, 39). The Indian Academy of Pediatrics (IAP) published guidelines on screen time and digital wellness in 2022. The guidelines label >1–2 h/day of screen time as “excessive” for 5–10-year-olds. For older adolescents, it is emphasized that screen time should not replace outdoor physical activity (4). The IAP guidelines for adolescents also recommend “a balance of screen time with at least one hour of outdoor physical activity (playtime), 8 to 9 hours of night-time sleep, and time for schoolwork, meals, hobbies, peer interaction, and family time”. Adolescents are advised to “ensure that screen use is not interfering with their academic performance, mental health, talent development, and acquisition of values” (4).

In our study, there were significant urban–rural differences among adolescents with more than 2 h of daily screen time. In urban schools, there were more male students, more students who spent fewer than 2 days a week engaged in outdoor physical activity, and a greater proportion of students with some/high needs across all SDQ scales (except the prosocial scale) than in rural schools. Nigg et al. have reported a decreasing trend in outdoor physical activity with increasing screen time exposure in both urban and rural areas (40). Guo et al. have reported a greater decline in physical activity and an increase in recreational screen time among rural adolescents compared with urban adolescents (41). Our findings highlight a clear relationship between rural residence and a higher frequency of outdoor physical activity compared to urban areas, emphasizing that urban students spend less time outdoors. As per the results reported by Fan et al. based on the Youth Risk Behavior Surveillance System, computer/video time exceeding 2 h was a significant risk factor for reduced physical activity (42). Although the association was not statistically significant, we found that overall, a greater proportion of students with a daily total screen time of more than 2 h engaged in less than 2 days per week of outdoor physical activity than those with less than 2 h of daily screen time.

Poor psychological health in the form of poor quality of life, perceived insufficient peer support, anxiety and depression (14, 35, 43, 44) have also been associated with screen time exposure. Research on children, teenagers, and adolescent age groups has shown that excessive screen time, usually greater than 2 h per day, is not only associated with psychological or behavioral problems but with more severe mental health problems such as depression, paranoia and suicidal ideation (11). Yu et al. studied 4,932 children and adolescents and found significant associations between excessive screen time (>2 h) and emotional, conduct, and peer problems, and their total difficulties scores on the SDQ (45). Tamura et al. used the SDQ and found significant associations between longer screen time duration and worse scores on hyperactivity, peer problems and prosocial behavior scales in 7- to 17-year-olds. They found that those who spent more than 2 h playing video games on weekdays had higher odds of problematic total difficulties scores (46). In our present study, the psychological needs assessment based on scores across all SDQ scales showed that a greater proportion of students with daily screen time exposure of >2 h had some/high psychological needs as compared to those with ≤2 h, being statistically significant for the hyperactivity scale. Since there are no other studies on Indian adolescents that have explored such associations and compared urban–rural scenarios, we have discussed our findings in light of studies conducted mainly in the Western world and other Asian countries.

Lastly, we would like to point to literature that reports that social media in itself does not inherently pose a threat to adolescent mental health. Agyapong-Okopu et al. argue that it is the problematic use of social media that causes harm with active versus passive online activity defining this relationship (47).

Based on our findings, especially those highlighting urban–rural differences, there is a clear need to develop tailor-made school-based interventions for Indian schools. Currently, as Indian schools prohibit students from carrying mobile phones to class, the interventions should incorporate parental involvement, as parents would have an important role to play in achieving a reduction in their children’s screen time at home.

## Limitations and strengths

Our study had several limitations. We did not collect data on the online content students consumed to distinguish between educational and recreational screen time, nor their screen time exposure on weekends or holidays. Additionally, parental screen time habits and parental sociodemographic details would have likely provided a better backdrop to this issue. We used the Strengths and Difficulties Questionnaire, which helped evaluate the emotional, conduct, hyperactivity, peer problems and prosocial behavior of our participants. We have not assessed depression, anxiety, or stress in these adolescents. Other possible factors which could be associated with psychological problems in this age group need to be considered through a more nuanced psychological assessment. We would also like to acknowledge the limited generalizability of our findings as they represent only a specific region in India.

The strengths of our study were the large sample size with a wide geographical coverage, urban–rural comparisons, and the use of self-reported screen time durations rather than reliance on parental reporting, which could be erroneous.

## Conclusion

The daily screen time exposure of both urban and rural adolescent students is high, with urban students and boys having longer screen time exposures than rural students and girls. Urban adolescents had less frequent engagement in outdoor physical activity than their rural counterparts. A greater proportion of students with longer than 2 h of daily screen time were overweight and obese as compared to those with less than 2 h of exposure. Students with longer screen time durations had some or high psychological needs.

Future studies examining the same research question in different cultural settings could highlight the variability of these findings across regions and countries. Also, studies focusing on the intricacies of urban and rural adolescents’ screen time behaviors such as difference between educational and recreational screen time, and the online content consumed are needed to tailor appropriate region-specific interventions.

## Data Availability

The raw data supporting the conclusions of this article will be made available by the authors, without undue reservation.
